# Inpatient Hospitalization Costs Associated with Birth Defects Among Persons of All Ages — United States, 2013

**DOI:** 10.15585/mmwr.mm6602a1

**Published:** 2017-01-20

**Authors:** Annelise C. Arth, Sarah C. Tinker, Regina M. Simeone, Elizabeth C. Ailes, Janet D. Cragan, Scott D. Grosse

**Affiliations:** ^1^Division of Congenital and Developmental Disorders, National Center on Birth Defects and Developmental Disabilities, CDC; ^2^Office of the Director, National Center on Birth Defects and Developmental Disabilities, CDC.

In the United States, major structural or genetic birth defects affect approximately 3% of live births (*1*) and are responsible for 20% of infant deaths (*2*). Birth defects can affect persons across their lifespan and are the cause of significant lifelong disabilities. CDC used the Healthcare Cost and Utilization Project (HCUP) 2013 National Inpatient Sample (NIS), a 20% stratified sample of discharges from nonfederal community hospitals, to estimate the annual cost of birth defect–associated hospitalizations in the United States, both for persons of all ages and by age group. Birth defect–associated hospitalizations had disproportionately high costs, accounting for 3.0% of all hospitalizations and 5.2% of total hospital costs. The estimated annual cost of birth defect–associated hospitalizations in the United States in 2013 was $22.9 billion. Estimates of the cost of birth defect–associated hospitalizations offer important information about the impact of birth defects among persons of all ages on the overall health care system and can be used to prioritize prevention, early detection, and care.

CDC used the HCUP 2013 NIS sponsored by the Agency for Healthcare Research and Quality (*3*). The NIS is a 20% stratified sample of discharges from nonfederal community hospitals and does not include rehabilitation and long-term care hospitals. Readmissions for the same person cannot be distinguished, and a person might be included in the data more than once, therefore these data cannot be used to study costs at an individual level. Patients who die during their hospitalization are included in the NIS. CDC included discharges among patients of all ages from January 1 through December 31, 2013. Birth defects were identified through *International Classification of Diseases, Ninth Revision, Clinical Modification* (ICD-9-CM) codes 740.00–759.9. CDC did not consider the following conditions to be birth defects: 1) persistent fetal circulation (747.83) or balanced autosomal translocation in a normal person (758.4), if they were the only codes in this range recorded for the discharge; and 2) patent ductus arteriosus (747.0) or ostium secundum type atrial septal defect (745.5; which includes patent foramen ovale as well as actual atrial septal defects) if they were the only birth defect codes for preterm infants or term infants aged <28 days. For infants aged <1 year, CDC classified acquired pyloric stenosis (537.0) as a birth defect; for persons aged ≥1 year, this was not considered a birth defect. Hospitalizations that included at least one discharge diagnosis with a birth defect ICD-9-CM code meeting these definitions were considered “birth defect–associated” hospitalizations. Eligible birth defect codes found in any diagnosis field (i.e., primary or any of 24 reported secondary fields) were analyzed for all birth defects combined, for categories of birth defects broadly defined by organ system (*4*), and for individual defects.

Cost was calculated as the product of facility fee charge, cost-to-charge ratio, and professional fee ratio. The cost-to-charge ratio is used to account for the difference in the amount billed (charge) and the payment received by hospitals (cost). CDC used the HCUP hospital-specific all-payer inpatient cost-to-charge ratio when available, or the weighted group average all-payer inpatient cost-to-charge ratio otherwise (*5*). Hospital charges represent the facility fees charged by hospitals and do not include the cost of physician services, which are billed separately. Professional fee ratios (PFR) provide the means to adjust charges to reflect the estimated cost of services by physicians, which often account for 20%–25% of the cost of a given hospital visit (*6*). CDC obtained PFR data for 2012 Medicaid and commercial insurance and assigned PFRs to discharges by diagnostic-related group (*6*). All discharges of patients aged ≥65 years were assigned commercial PFRs (because Medicare reimburses at a similar rate to commercial insurance), whereas discharges of patients aged <65 years were assigned PFRs based on the coded payer. Data analysis was performed with SAS 9.4 using survey procedures to account for sampling design. The weighted number of discharges with a birth defect diagnosis code were totaled. The mean, median, and total cost were calculated and stratified by organ system and individual defects.

The total weighted cost for birth defect–associated hospitalizations was $22,946,158,457 (Table 1), representing 5.2% of total costs for all hospital discharges. The costs for birth defect–associated hospitalizations were highest among patients aged <1 year ($8,901,015,375) compared with other age groups (Table 1). Among admissions of all patients aged <1 year, birth defect–associated hospitalizations represented 35.0% of total costs. For patients aged 1–5 years, the cost of birth defect–associated hospitalizations was $1,532,487,122, representing 6.7% of total birth defect–associated hospitalization costs. The median cost for birth defect–associated hospitalizations was lowest among patients aged <1 year ($2,126) and highest among patients aged ≥65 years ($13,270) (Table 1).

**TABLE 1:** Weighted estimates for numbers of hospitalizations with at least one birth defect–associated discharge diagnosis,* by age group — National Inpatient Sample, United States, 2013

Age (yrs)	No. (%)^†^	(95% CI)	Total cost ($) (%)^†^	(95% CI)	Mean cost ($) (95% CI)	Median cost ($) (IQR)
<1	417,495 (39.3)	(395,092–439,897)	8,901,015,375 (38.8)	(7,671,927,338–10,130,100,000)	21,320 (19,064–23,575)	2,126 (1,108–9,835)
1–5	65,485 (6.2)	(54,843–76,127)	1,532,487,122 (6.7)	(1,192,717,252–1,872,256,992)	23,402 (21,311–25,492)	10,218 (4,958–22,632)
6–18	73,730 (6.9)	(62,783–84,676)	1,980,819,467 (8.6)	(1,579,567,292–2,382,071,642)	26,866 (24,550–29,181)	12,971 (6,235–26,735)
19–64	322,480 (30.4)	(311,315–333,645)	6,640,681,622 (28.9)	(6,281,399,417–6,999,963,827)	20,593 (20,013–21,171)	11,713 (6,251–24,364)
≥65	181,815 (17.1)	(175,683–187,946)	3,891,154,870 (17.0)	(3,711,725,515–4,070,584,226)	21,402 (20,852–21,950)	13,270 (7,437–25,941)
**Total**	**1,061,004**	**(1,015,274–1,106,733)**	**22,946,158,457**	**(20,894,139,517–24,998,177,397)**	**21,626 (20,415–22,837)**	**8,366 (2,700–20,920)**

**Abbreviations:** CI = confidence interval; IQR = interquartile range.

* Any primary or secondary (up to 25 total) *International Classification of Diseases, Ninth Revision*, *Clinical Modification* (ICD-9-CM) discharge diagnosis codes 740–759, with the following exceptions: 1) when persistent fetal circulation (747.83) or balanced autosomal translocation in a normal person (758.4) were the only codes in this range recorded for the discharge; or 2) when patent ductus arteriosus (747.0) or ostium secundum type atrial septal defect (745.5, which includes patent foramen ovale as well as actual atrial septal defects) were the only defects coded in a preterm infant or an infant aged <28 days. Infants aged <1 year were classified as having a birth defect if they had acquired pyloric stenosis coded (537.0).

^†^ Because of weighting and rounding, estimates might not sum.

Among the organ systems considered (Table 2), cardiovascular defects accounted for the largest percentage of birth defect–associated hospitalizations (14.0%), and the highest total cost, approximately $6.1 billion (26.6% of total birth defect–associated hospitalization costs). Within cardiovascular defects, critical congenital heart defect–associated hospitalizations had the highest mean and median cost of the birth defect categories considered ($79,011 and $29,886, respectively). Central nervous system defects accounted for the second most frequent birth defect–associated hospitalizations (6.2%), with a total cost of approximately $1.7 billion. Among noncardiovascular defects, eye defect–associated hospitalizations had the highest mean cost ($44,441), and ear defects had the highest median cost ($11,349). The specific birth defect with the highest median hospitalization cost was interrupted aortic arch (median = $76,109; interquartile range [IQR] = $14,893–$170,601) (Figure).

**TABLE 2:** Weighted estimates for the number, total cost, mean cost, and median cost of birth defect–associated hospitalizations by organ system — National Inpatient Sample, 2013

Birth defect category*	No. (%)	(95% CI)	Total cost ($) (95% CI)	Mean cost ($) (95% CI)	Median cost ($) (IQR)
Central nervous system^†^	65,509 (6.2)	(59,971–71,048)	1,651,098,167 (1,445,247,680–1,856,948,653)	25,203 (23,727–26,680)	10,559 (5,215-22,859)
CNS: no congenital hydrocephalus^§^	54,444 (5.1)	(49,823–59,066)	1,280,902,711 (1,111,540,696–1,450,264,725)	23,526 (22,040–25,012)	10,453 (5,209-22,397)
Eye^¶^	2,174 (0.2)	(1,903–2,446)	96,660,092 (67,765,860–125,554,325)	44,441 (33,364–55,518)	10,341 (3,959-35,908)
Ear**	1,534 (0.1)	(1,249–1,820)	36,116,559 (22,459,211–49,773,908)	23,528 (16,564–30,493)	11,349 (3,118-24,017)
Cardiovascular^††^	148,184 (14.0)	(137,188–159,180)	6,100,303,945 (5,200,878,936–6,999,728,954)	41,166 (37,630–44,703)	14,552 (6,450-39,316)
CV: critical congenital^§§^	29,349 (2.8)	(24,583–34,116)	2,318,986,684 (1,774,146,902–2,863,826,466)	79,011 (71,017–87,005)	29,886 (8,367-82,363)
CV: no atrial septal defect ^¶¶^	76,739 (7.2)	(68,370–85,109)	4,138,104,641 (3,351,220,890–4,924,988,393)	53,923 (48,739–59,107)	16,415 (5,105-53,284)
CV: no chromosomal defect***	136,374 (12.9)	(126,819–145,930)	5,467,287,739 (4,689,674,535–6,244,900,944)	40,090 (36,687–43,492)	14,236 (6,404-38,059)
Orofacial^†††^	18,439 (1.7)	(16,045–20,834)	412,768,370 (336,268,066–489,268,673)	22,384 (19,851–24,917)	9,051 (3,780-17,012)
Gastrointestinal^§§§^	31,639 (3.0)	(28,416–34,863)	1,118,964,869 (931,459,727–1,306,470,010)	35,365 (32,012–38,718)	9,737 (5,656-23,686)
Genitourinary^¶¶¶^	46,189 (4.4)	(44,287–48,092)	899,615,075 (808,860,197–990,369,952)	19,476 (17,966–20,986)	7,446 (2,374-17,705)
Musculoskeletal****	27,794 (2.6)	(25,664–29,925)	1,034,300,771 (856,588,012–1,212,013,530)	37,211 (32,841–41,582)	10,370 (2,391-28,792)
Chromosomal^††††^	48,464 (4.6)	(45,339–51,590)	1,145,462,342 (980,305,170–1,310,619,515)	23,634 (21,292–25,977)	9,067 (4,487-20,743)

**Abbreviations:** CI = confidence interval; CNS = central nervous system; CV = cardiovascular; IQR = interquartile range.

* Includes primary or any of 24 secondary *International Classification of Diseases, Ninth Revision, Clinical Modification* (ICD-9-CM) discharge diagnosis codes.

^†^ Anencephalus (740.0, 740.1), spina bifida without anencephalus (741.00–741.03, 741.90–741.93 without 740.0 or 740.1), congenital hydrocephalus without spina bifida (742.3 without 741.00–741.03, 741.90–741.93), encephalocele (742.0), microcephalus (742.1), or holoprosencephaly (742.2).

^§^ Same as central nervous system but does not include birth defect–associated hospitalizations where congenital hydrocephalus is the only defect.

^¶^ Anophthalmia/microphthalmia (743.00, 743.10, 743.11, 743.12), congenital cataract (743.30, 743.31, 743.32, 743.33, 743.34), or aniridia (743.45).

** Anotia/microtia (744.01, 744.23).

^††^ Common truncus (745.0), transposition of the great arteries (745.10, 745.12, 745.19), tetralogy of Fallot (745.2), ventricular septal defect (745.4), atrial septal defect (745.5 except when it was the only defect coded in a preterm infant or an infant <28 days old), endocardial cushion defect (745.60, 745.61, 745.69), pulmonary valve atresia and stenosis (746.01, 746.02), tricuspid valve atresia and stenosis (746.1), Ebstein anomaly (746.2), aortic valve stenosis (746.3), hypoplastic left heart syndrome (746.7), patent ductus arteriosus (747.0, except when it was the only defect coded in a preterm infant or an infant <28 days old), coarctation of aorta (747.10), double outlet right ventricle (745.11), interrupted aortic arch (747.11), single ventricle (745.3), or total anomalous pulmonary venous connection (747.41).

^§§^ Common truncus (745.0), transposition of the great arteries (745.10), tetralogy of Fallot (745.2), pulmonary valve atresia (746.01), tricuspid valve atresia and stenosis (746.1), Ebstein anomaly (746.2), hypoplastic left heart syndrome (746.7), coarctation of aorta (747.10), double outlet right ventricle (745.11), interrupted aortic arch (747.11), single ventricle (745.3), or total anomalous pulmonary venous connection (747.41.)

^¶¶^ Same as cardiovascular but does not include birth defect–associated hospitalizations where atrial septal defect is the only defect.

*** Same as cardiovascular but does not include hospitalizations that include chromosomal abnormalities (trisomy 21 (758.0), trisomy 13 (758.1), trisomy 18 (758.2), Cri-du-chat syndrome (758.31), 22q11 deletion syndrome (758.32), Turner syndrome (758.6), Klinefelter syndrome (758.7), other chromosomal conditions (758.33, 758.39, 758.5, 758.81, 758.89, 758.9).

^†††^ Cleft lip with cleft palate (749.20–749.25), cleft palate without cleft lip (749.00–749.04), cleft lip (749.10–749.14), or choanal atresia (748.0).

^§§§^ Esophageal atresia (750.3), rectal and large intestinal atresia/stenosis (751.2), pyloric stenosis (750.5 among all ages; 537.0 among infants aged <1 year), Hirschsprung disease (751.3), biliary atresia (751.61), or small intestinal atresia (751.1).

^¶¶¶^ Renal agenesis/hypoplasia (753.0), bladder exstrophy (753.5), hypospadias (752.61), epispadias (752.62), congenital posterior urethral valves (753.6).

**** Limb reduction deformity (755.20-755.39), gastroschisis (756.73), omphalocele (756.72), congenital hip dislocation (754.30, 754.31, 754.35), diaphragmatic hernia (756.6), clubfoot (754.51, 754.70).

^††††^ Trisomy 21 (758.0), trisomy 13 (758.1), trisomy 18 (758.2), 22q11 deletion syndrome (758.32), Turner syndrome (758.6).

**FIGURE:**
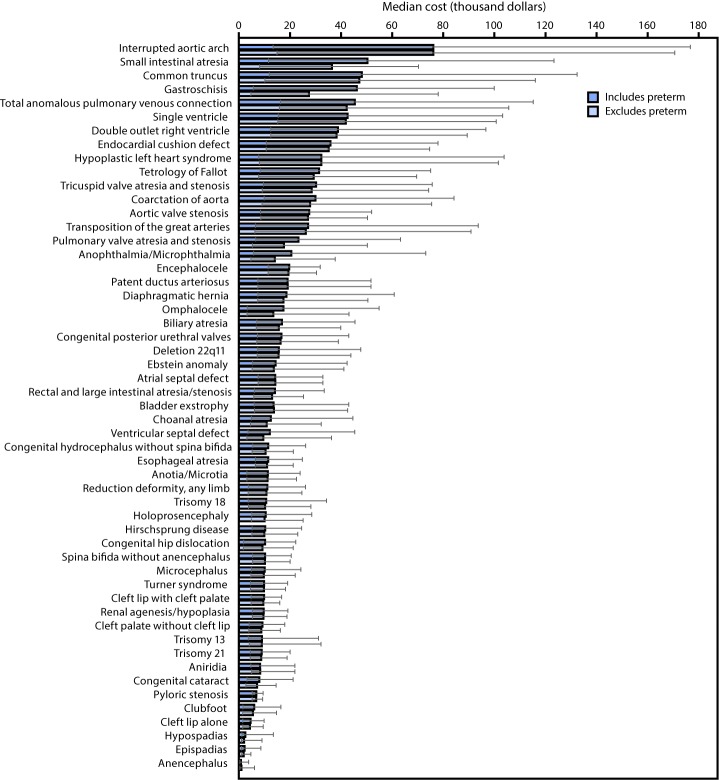
Weighted estimated median cost and interquartile range of birth defect–associated hospitalizations, by specific birth defect,*^,†^ — National Inpatient Sample, 2013 * *International Classification of Diseases, Ninth Revision,* *Clinical Modification* (ICD-9-CM) codes for each birth defect: anencephalus (740.0, 740.1); spina bifida without anencephalus (741.00, 741.01, 741.02, 741.03, 741.90, 741.91, 741.92, 741.93 without 740.0 or 740.1); congenital hydrocephalus without spina bifida (742.3 without 741.00-741.03, 741.90-741.93); encephalocele (742.0); microcephalus (742.1); holoprosencephaly (742.2); anophthalmia/microphthalmia (743.00, 743.10, 743.11, 743.12); congenital cataract (743.30, 743.31, 743.32, 743.33, 743.34); aniridia (743.45); anotia/microtia (744.01, 744.23); common truncus (745.0); transposition of great arteries (745.10, 745.12, 745.19); tetralogy of Fallot (745.2); ventricular septal defect (745.4); atrial septal defect (745.5, except when it was the only defect coded in a preterm infant or an infant <28 days old); endocardial cushion defect (745.60, 745.61, 745.69); pulmonary valve atresia and stenosis (746.01, 746.02); tricuspid valve atresia and stenosis (746.1); Ebstein anomaly (746.2); aortic valve stenosis (746.3); hypoplastic left heart syndrome (746.7); patent ductus arteriosus (747.0, except when it was the only defect coded in a preterm infant or an infant <28 days old); coarctation of aorta (747.10); double outlet right ventricle (745.11); interrupted aortic arch (747.11); single ventricle (745.3); total anomalous pulmonary venous connection (747.41); cleft palate without cleft lip (749.00, 749.01, 749.02, 749.03, 749.04); cleft lip with cleft palate (749.20, 749.21, 749.22, 749.23, 749.24, 749.25); cleft lip alone (749.10, 749.11, 749.12, 749.13, 749.14); choanal atresia (748.0); esophageal atresia/tracheoesophageal fistula (750.3); rectal and large intestinal atresia/stenosis (751.2); pyloric stenosis (750.5 among all ages; 537.0 among infants aged <1 year); Hirschsprung disease (751.3); biliary atresia (751.61); small intestinal atresia/stenosis (751.1); renal agenesis/hypoplasia (753.0); bladder exstrophy (753.5); hypospadias (752.61); epispadias (752.62); congenital posterior urethral valves (753.6); reduction deformity (755.20-755.39); gastroschisis (756.73); omphalocele (756.72); congenital hip dislocation (754.30, 754.31, 754.35); diaphragmatic hernia (756.6); clubfoot (754.51, 754.70); trisomy 13 (758.1); trisomy 21 (758.0); trisomy 18 (758.2); 22q11.2 deletion syndrome (758.32); Turner syndrome (758.6). ^†^ Preterm birth was defined as <37 weeks gestational age (ICD-9-CM codes 765.00–.09, 765.10–.19, 765.21–.28, or Diagnosis Related Group codes 791–792.

Among birth defect–associated hospitalizations, 11.8% had a primary birth defect ICD-9-CM code. The total estimated cost for those hospitalizations was $5,043,781,895 (95% confidence interval [CI] = $4,184,620,375–$5,902,943,416), accounting for 1.1% of total hospital costs and 22.0% of birth defect–associated hospitalization costs when all discharge diagnoses were included. Among the birth defect types examined using only the primary birth defect ICD-9-CM codes, hypoplastic left heart syndrome had the highest mean cost ($164,994; 95% CI = $133,224–$196,763) and interrupted aortic arch had the highest median cost ($119,303; IQR = $68,223–$189,344).

After excluding discharges with ICD-9-CM or Diagnosis Related Group codes indicating preterm birth, the total estimated cost for birth defect–associated hospitalizations was $18,884,865,845 (95% CI = $17,185,471,370–$20,584,260,320) or 82.3% of total costs of all birth defect–associated discharges.

## Discussion

CDC’s analysis of NIS data indicates that the annual cost of hospitalizations that included a birth defect discharge diagnosis code in 2013 was $22.9 billion. Although birth defect–associated hospitalizations accounted for 3.0% of all hospitalizations, they accounted for 5.2% of total hospital costs, highlighting the disproportionately high costs of treating patients with these conditions. The share of costs was especially high for infants, accounting for 35.0% of total hospitalization costs for children aged <1 year. Across all ages, costs were particularly high for hospitalizations associated with cardiovascular defects, which accounted for approximately 14.0% of birth defect–associated hospitalizations but 26.6% of birth defect–associated costs.

In a previous analysis of 2004 HCUP data, the total cost of birth defect–associated hospitalizations was estimated at $2.6 billion (*7*). This estimate was based only on primary ICD-9-CM discharge diagnosis codes. Inclusion of only primary diagnosis codes in this analysis resulted in an estimate of $5.0 billion. However, estimates based only on the primary ICD-9-CM codes are likely to be an underestimate of costs, because birth is often coded as the principal diagnosis for birth hospitalizations (*8*), and because birth defects might be important factors contributing to hospitalizations associated with other primary diagnosis codes.

The findings in this report are subject to at least five limitations. First, use of all diagnosis codes might have overestimated costs because the coded birth defect might have been incidental to the reason for the hospitalization. Conversely, birth defects that influence conditions leading to hospitalization might be less likely to be coded as a person ages. Second, the primary analysis included preterm infants, who have higher associated hospitalization costs (*9*), potentially leading to an overestimate of cost. Although preterm birth is more common in infants with birth defects (*10*), the extent to which hospitalization costs are attributable to preterm birth, rather than the birth defect, cannot be estimated with these data. Third, some children had more than one birth defect diagnosis; attributing the cost of hospitalization to each defect independently in these children might have resulted in an overestimate of the cost of one or more of the individual defects. Fourth, although NIS data are routinely used for research, their source data were originally created for billing purposes and diagnoses are not validated, which might have led to an over- or underestimate of average costs. Finally, the cost-to-charge ratios used in this analysis were based on aggregated hospital data and were not specific to the departments or treatments more likely to be used for birth defect hospitalization, which might have affected the cost estimate in either direction.

By estimating the cost of birth defect–associated hospitalizations, both researchers and policy makers can be more informed of the impact of birth defects on the health care system and can use this knowledge to motivate change through prevention, early detection, and care throughout the lifespan of affected persons.

**Summary**

**What is already known about this topic?**

Major structural or genetic birth defects affect approximately 3% of live births and are responsible for 20% of infant deaths.

**What is added by this report?**

Analysis of 2013 hospital discharge data found that birth defect–associated hospitalizations accounted for 3.0% of all hospitalizations and 5.2% of total hospital costs. The estimated annual cost of U.S. hospitalizations that included a birth defect code among any discharge diagnosis was $22.9 billion, whereas the estimated cost based on having a primary birth defect discharge diagnosis code was $5.0 billion. When birth defects among any diagnosis code were included, but preterm delivery codes were excluded, the total estimated cost was $18.9 billion.

**What are the implications for public health practice?**

Estimates of the cost of birth defect–associated hospitalizations offer important information on the impact of birth defects on the overall health care system and can be used to prioritize prevention measures.
